# A human cancer-predisposing polymorphism in Cdc25A is embryonic lethal in the mouse and promotes ASK-1 mediated apoptosis

**DOI:** 10.1186/1747-1028-6-4

**Published:** 2011-02-10

**Authors:** El Mustapha Bahassi, Moying Yin, Susan B Robbins, Ya-Qin Li, Deborah G Conrady, Zhenyu Yuan, Rhett A Kovall, Andrew B Herr, Peter J Stambrook

**Affiliations:** 1 Division of Hematology and Oncology, Department of Internal Medicine, University of Cincinnati, 231 Albert Sabin Way, Cincinnati, OH 45267-0562, USA; 2 Department of Molecular Genetics, Biochemistry, and Microbiology, University of Cincinnati College of Medicine, Cincinnati, Ohio 45267, USA

## Abstract

**Background:**

Failure to regulate the levels of Cdc25A phosphatase during the cell cycle or during a checkpoint response causes bypass of DNA damage and replication checkpoints resulting in genomic instability and cancer. During G1 and S and in cellular response to DNA damage, Cdc25A is targeted for degradation through the Skp1-cullin-β-TrCP (SCF^β-TrCP^) complex. This complex binds to the Cdc25A DSG motif which contains serine residues at positions 82 and 88. Phosphorylation of one or both residues is necessary for the binding and degradation to occur.

**Results:**

We now show that mutation of serine 88 to phenylalanine, which is a cancer-predisposing polymorphic variant in humans, leads to early embryonic lethality in mice. The mutant protein retains its phosphatase activity both *in vitro *and in cultured cells. It fails to interact with the apoptosis signal-regulating kinase 1 (ASK1), however, and therefore does not suppress ASK1-mediated apoptosis.

**Conclusions:**

These data suggest that the DSG motif, in addition to its function in Cdc25A-mediated degradation, plays a role in cell survival during early embyogenesis through suppression of ASK1-mediated apoptosis.

## Introduction

When DNA is damaged or when blocks to DNA replication occur, cells activate an extensive network of signaling pathways that promote cell cycle arrest and DNA repair [1]. Cell cycle arrest is mediated by checkpoints in which the activities of cyclin-dependent kinases (Cdks) are inhibited. Cell cycle arrest presumably provides time for damage to be repaired and contributes to the preservation of genomic stability and reduction of risk for diseases such as cancer. There are two well established mechanisms by which Cdks are inhibited in response to DNA damage in mammals. The first involves activation of the Cdk inhibitor p21^Cip ^through activation of p53 [2]. The second is through inactivation of the Cdc25 family of phosphatases. The most fully understood mechanism regulating the level of a phosphatase in this family is the proteasome-mediated degradation of Cdc25A [3-5].

Cdc25A phosphatase is an essential activator of cell cycle progression and its expression is tightly regulated at many levels, including transcriptional activation, reversible phosphorylation, protein-protein interaction and ubiquitin-mediated degradation [6-9]. Ubiquitin-dependent degradation of Cdc25A is a major mechanism for damage-induced S-phase checkpoint arrest. Two ubiquitin ligases, the Skp1-cullin-β-TrCP (SCF^β-TrCP^) complex and the anaphase-promoting complex (APC^Cdh1^), participate in Cdc25A turnover. The APC/C proteasome complex helps regulate Cdc25A at the exit of mitosis while SCF^β-TrCP ^regulates the abundance of Cdc25A in S phase and G2 [8,10-13]. When DNA is damaged or when cells respond to stalled replication forks, ATM and ATR protein kinases are activated leading to subsequent activation of Chk1 and Chk2 and to hyperphosphorylation of Cdc25A. This cascade of events stimulates SCF-mediated ubiquitinylation of Cdc25A and its proteolysis [3,5], contributing to cell cycle arrest. Failure to regulate Cdc25A levels compromises checkpoint arrest and can result in enhanced DNA damage [3-5,14,15]. Overexpression of Cdc25A, which frequently occurs in multiple tumor types [16], leads to accelerated entry of cells into S-phase [17] and mitosis [3].

The ubiquitinylation-mediated degradation of Cdc25A is associated with phosphorylation of Cdc25A at two residues within the β-TrCP docking site (DSG motif). The DSG docking site of Cdc25A is comprised of serines 79, 82 and 88, and the absence of their phosphorylation is sufficient to abolish β-TrCP binding and interfere with Cdc25A degradation [10]. Several kinases have been reported to phosphorylate the DSG motif and to target Cdc25A for SCF^β-TrCP^-mediated degradation [18-20]. The NIMA related kinase 11 (NEK11) has been identified as a kinase that specifically phosphorylates Cdc25A on serines 82 and 88 in cultured cells. This phosphorylation is important for subsequent ubiquitinylation and proteasome-mediated degradation [19]. The existence of an S88F polymorphic variant in humans [21] that elevates the risk for cancer [22] underscores the importance of serine 88 in cell function, behavior and disease. Its importance appears to have been conserved in evolution since the same serine to phenylalanine mutation in *C. elegans *at a serine equivalent to human serine 88 is a gain of function mutation that causes deregulated intestinal cell hyper-proliferation and hyperplasia [23].

To better understand the significance of Cdc25A phosphorylation within the DSG domain in cell cycle regulation and DNA damage response, we generated a knock-in mouse model with a substitution of phenylalanine for serine at residue 88. The expectation was that cells from mice harboring this Cdc25A variant would be less susceptible to SCF^β-TrCP^-mediated degradation. Inhibition of Cdc25A degradation in turn should lead to its accumulation, accompanied by bypass of DNA damage and replication checkpoints, enhanced DNA damage and increased risk of cancer. In the mouse, we find that this mutation, when homozygous, produces early fetal lethality, indicating that the Cdc25A DSG motif and serine 88 phosphorylation may also have a role in early embryogenesis. Homozygous mutant embryos that persist display an altered morphology with extensive cellular degeneration and die at or prior to embryonic day 3.5. Circular dichroism (CD) studies revealed that the S88F substitution in Cdc25A protein had no major effect on protein secondary structure that would account for the observed phenotype. The mutant protein retains phosphatase activity but, unlike the wildtype Cdc25A, the S88F mutant fails to interact with the apoptosis signal-regulating kinase-1 (ASK1) facilitating apoptosis mediated by ASK1. These data demonstrate that the DSG domain of Cdc25A has a role in cell survival during early embryogenesis that is separate from its role in Cdc25A ubiquitin-mediated degradation in response to DNA damage.

## Results

### Serines 82 and 88 are required for efficient degradation of Cdc25A following DNA damage

**Figure 1:**
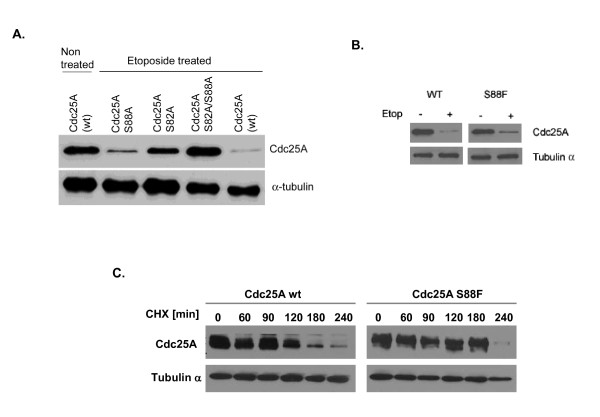
**Cdc25A S82 and S88 are required for full degradation of the protein following DNA damage**. HT1080 cells were transfected with FLAG-Cdc25A-wt, FLAG-Cdc25A-S82A, FLAG-Cdc25A-S88A, FLAG-Cdc25A-S82A/S88A or FLAG-Cdc25A-S88F. **A) **When cells were treated with 40 μM etoposide for 12 hours, FLAG-Cdc25A-wt was efficiently degraded while mutant Cdc25A S82A and mutant S88A were partially degraded. The double mutant S82A/S88A was completely resistant to degradation following DNA damage. **B) **Similar to FLAG-Cdc25A-S88A, FLAG-Cdc25A-S88F was partially degraded following treatment with etoposide (40 μM) for 12 hours. **C) **Exponentially growing cells transfected with either Flag-Cdc25A or Flag-Cdc25A-S88F were treated with cycloheximide (50 μg/mL) alone to block the protein synthesis. At the indicated time intervals cell samples were collected and Cdc25A expression was detected by western blot using Flag-M2 antibody. α-tubulin levels are shown as loading controls. Both Flag-M2 and α-tubulin monoclinal antibodies were purchased from Sigma Aldrich.

To confirm the role of the DSG domain in Cdc25A degradation following DNA damage, cDNA encoding Cdc25A was subjected to site-directed mutagenesis to replace serine residues 82 and 88 with an alanine either individually or together. The degree to which wildtype or mutant Cdc25A was degraded was assessed following transfection of HEK-293 cells and treatment with the radiomimetic etoposide (Figure 1A). When both serine residues were replaced with an alanine, Cdc25A protein was rendered fully resistant to SCF^β-TrCP^-mediated degradation following etoposide treatment. Substitution of either serine 82 or 88 to alanine individually was sufficient to confer partial resistance to degradation of Cdc25A following DNA damage (Figure 1A). Substitution of serine 88 to phenylalanine also rendered Cdc25A protein partially resistant to degradation following treatment with etoposide (Figure 1B). The half-life of Cdc25A wild type and S88F mutant proteins in the absence of treatment with a DNA damaging agent was also investigated. Cells were pretreated with cycloheximide to inhibit *de novo *protein synthesis, and at the indicated time intervals cells were collected, lysed and the Cdc25A protein detected used Flag-M2 antibody (Figure 1C). The half-life of the Cdc25A S88F mutant protein was found to be higher than the wild type protein (Figure 1C).

### Cdc25A S88F induces early embryonic lethality in mice

**Figure 2:**
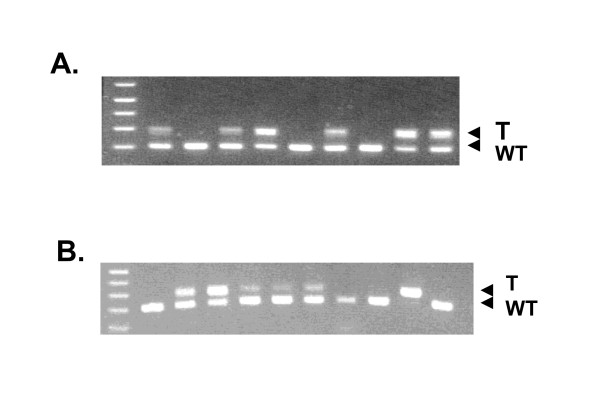
**Mutation of serine 88 to phenylalanine in Cdc25A induces early embryonic lethality in mice**. **A) **Heterozygous mutant mice carrying a serine 88 to phenylalanine mutation were mated and the resulting pups were genotyped. No homozygous mutant mice were obtained in the progeny. **B) **Further analysis shows that the earliest live homozygous embryos were obtained at the blastocyst stage (day E3.5).

To better understand the roles of the Cdc25A DSG domain in development and oncogenesis, we generated a *Cdc25A*-S88F mouse line by standard gene targeting in embryonic stem cells. A point mutation in exon 3 leading to substitution of serine 88 with phenylalanine was introduced using a floxed neomycin-resistant gene cassette. Although heterozygous mice are viable and fertile, no mice homozygous for *Cdc25A *S88F were born. Table 1 shows that out of 277 live pups from matings between heterozygotes, there were no homozygous mutants (0%) while 23% were wildtype and 77% were heterozygous for mutant Cdc25A (Table 1, Figure 2A). To determine when during gestation the embryos die, embryos were genotyped at days E12.5, E10.5, E7.5, E5.5, E3.5, E2.5 and E1.5. The latest time at which any homozygous mutant embryos were detected was at the blastula stage (E3.5) (Figure 2B and Table 1). Analysis of embryo morphology indicated that embryos homozygous for the mutant allele die at or before the blastocyst stage (E3.5 embryos). Only 7% of the embryos at this stage were homozygous for the mutant allele rather than the expected 25% (Table 1). The same was true at the morula stage (E2.5) and essentially at the cleavage stage (E1.5) where 0% were observed (Table 1). The data suggest that the variant allele, when homozygous, manifests at or before early cleavage.

**Table 1:** Genotype distribution in the pups and embryos from Cdc25A heterozygous crosses

*N*	Age (day)	+/+	+/-	-/-
277	Pups <22	64 (23)	213 (77)	0
19	e12.5	2 (11)	17 (89)	0
45	e3.5	18 (40)	24 (53)	3 (7)
35	e2.5	13 (37)	20 (57)	2 (6)
93	e1.5	47 (51)	46 (49)	0

*Expected*		*(25)*	*(50)*	*(25)*

All parents were +/-. *N *represents the total number of pups or embryos of each age. The numbers in parentheses indicate the percentage of pups or embryos of a given genotype based on the total number of that age.

### Expression of Cdc25A S88F is lethal to early embryos and cultured cells

**Figure 3:**
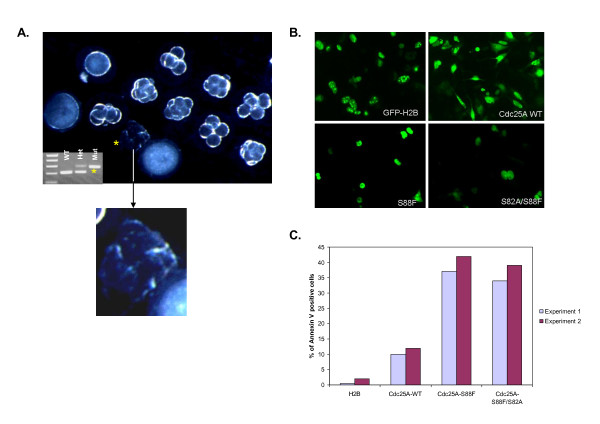
**Cells and embryos expressing serine 88 to phenylalanine mutation undergo cell death**. **A) **Homozygous embryos for the S88F mutation at day E3.5 appeared blebby and shrunken, indicative of cell death. An increased magnification of mutant cells undergoing apoptosis is shown. **B) **HT1080 cells transfected with GFP-Cdc25A-wt, GFP-Cdc25A-S88F, GFP-Cdc25-S88F/S82A or GFP-H2B control show that GFP-Cdc25A-S88F transfected cells round up and undergo cell death within 16 to 18 hours post transfection. **C) **Annexin V staining of HT1080 cells transfected with the same constructs as in B. The data from two independent experiments are shown.

To confirm that the S88F mutation leads to early embryonic lethality, we examined the morphology of early cleavage embryos and morulae that were characterized by PCR as homozygous for the mutant allele. The example in figure 3A shows that the only S88F homozygous embryo in the field (marked by an asterisk) was shrunken with bleb-like protuberances, suggestive of apoptotic cell death. Wildtype and heterozygous embryos appeared normal. To assess whether the S88F mutant protein by itself was sufficient to promote cell death, HT1080 cells were transfected with plasmids encoding wildtype Cdc25A, or mutant Cdc25A S88F or S82A/S82F proteins, and the cells were assayed for cell survival. A plasmid encoding a GFP-H2B fusion protein served as control. Ectopic expression of Cdc25A S88F and Cdc25A S82A/S88F led to rapid cell shrinkage and rounding, similar to what was seen in homozygous mutant embryos. Cell death occurred within 16 hours post transfection (Figure 3B). In contrast, transfection with plasmids encoding Cdc25A wildtype and GFP-H2B did not display any characteristics of cell death. Annexin V staining at 16 hours post transfection showed that Cdc25A mutants underwent cell death by apoptosis (Figure 3C).

### The S88F substitution confers only subtle conformational changes on Cdc25A

**Figure 4:**
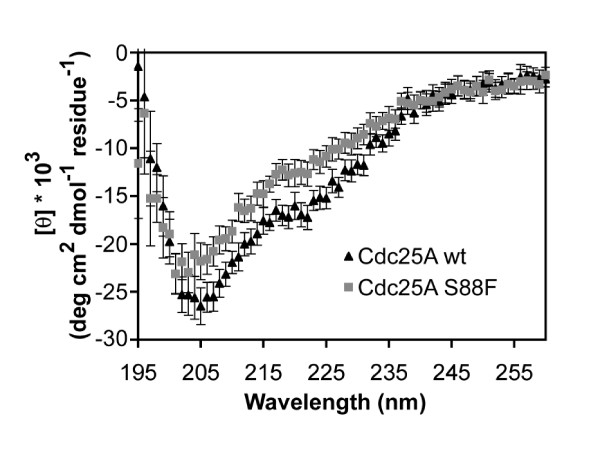
**Comparison of Cdc25A-WT and Cdc25A S88F mutant secondary structures**. The secondary structure content of Cdc25A-wt and the S88F mutant were compared by far-UV circular dichroism (CD). The CD spectra of the two proteins were similar, although they revealed small but significant differences.

One concern is that the S88F substitution causes a major conformational change in the protein that eliminates its phosphatase activity, which is essential for cell cycle progression. To determine whether this substitution significantly alters its secondary structure, Cdc25A-wt and Cdc25A S88F were compared by far-UV circular dichroism (CD). The CD spectra of the two proteins were similar, although they revealed small but significant differences (Figure 4). The similarity of the curves and the comparable secondary structure of the two samples suggest that the serine to phenylalanine substitution does not lead to large-scale structural changes. Deconvolution of the data suggests a transition to a slightly more β-strand-rich secondary structure content in the mutant protein (Table 2).

**Table 2:** Deconvolution of Cdc25A far-UV CD spectra using CDSSTR.

Sample	Helix	Strand	Turns	Coil	Total	NRMSD
Cdc25A-wt	8%	34%	23%	34%	99%	0.106
Cdc25A S88F	6%	40%	22%	31%	99%	0.107

### Cdc25A S88F retains phosphatase activity *in vitro *and in cultured cells

**Figure 5:**
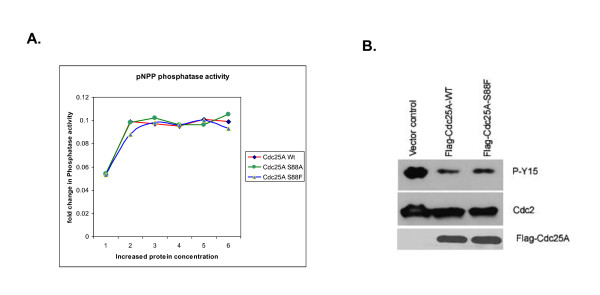
**Cdc25A S88F mutant retains its phosphatase activity both *in vitro *and *in vivo***. **A) **GST-Cdc25A-wt, GST-Cdc25A-S88A and GST-Cdc25A-S88F were assayed by hydrolysis of pNPP *in vitro*. No major effect on Cdc25A mutants was observed compared with wildtype. **B) **HT1080 cells were transfected with GFP-Cdc25A-WT, GFP-Cdc25A-S88F or GFP-H2B control; 12 hours post transfection, lysates from the transfected cells were assayed for Cdc2-Y15 phosphorylation. Cells equally transfected with either wildtype or mutant Cdc25A show similar levels of Y15 phosphorylation 12 hours post transfection. Anti-GFP antibody (a mixture of two monoclonal antibodies) was purchased from Roche Applied Sciences. Cdc2 and Cdc2-Y15 antibodies were purchased from Santa Cruz Biotech. (Santa Cruz, CA).

To directly assess the activity of the S88F variant, the phosphatase activities of Cdc25A S88F and wildtype Cdc25A were compared *in vitro *and in cultured cells. To measure relative phosphatase activities *in vitro*, the activities of wildtype Cdc25A, Cdc25A S88F and a Cdc25A S88A mutant were assayed by hydrolysis of 4-nitrophenol phosphate (pNPP). The phosphatase activity of Cdc25A S88F mutant is similar to that of wildtype Cdc25A and Cdc25A S88A, indicating that the serine to phenylalanine substitution does not inactivate Cdc25A phosphatase activity *in vitro *(Figure 5A). To confirm that Cdc25A S88F retains its phosphatase activity, cells were transfected with cDNAs encoding either wildtype Cdc25A or Cdc25A S88F and the level of Cdk1 (cdc2) dephosphorylation was monitored by assessing the level of phosphorylated (Y15) Cdk1, a key target of Cdc25 phosphatases. The level of Y15 phosphorylation was reduced following cell transfection with either Cdc25A-wt or Cdc25A S88F mutant compared with a GFP-H2B control, indicating that Cdc25A S88F mutant retains its phosphatase activity in cultured cells (figure 5B).

### Cdc25A S88F loses its interaction with ASK1 and fails to suppress the stress-inducible ASK1-mediated apoptotic pathway

**Figure 6:**
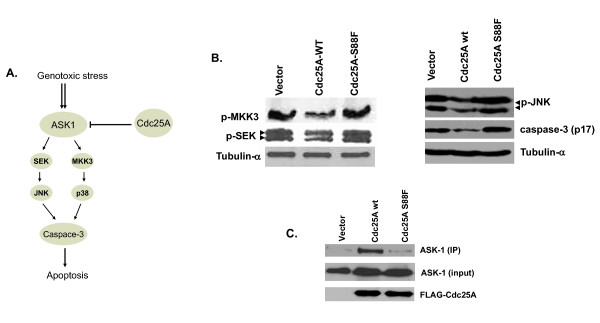
**Loss of interaction between Cdc25A S88F mutant and ASK1**. **A) **A schematic representation showing ASK1/Cdc25A interaction leading to suppression of the ASK1 apoptotic pathway. **B) **Suppression of the ASK1-mediated apoptotic downstream effectors by overexpression of Cdc25A-wt but not Cdc25A S88F mutant. **C) **Immunoprecipitation of ASK1 using either FLAG-Cdc25A-WT or FLAG-Cdc25A-S88F shows that S88F mutant fails to interact with ASK1. ASK-1, p-SEK, p-MKK3, p-JNK and Caspase-3 antibodies were all from Santa Cruz Biotech. (Santa Cruz, CA).

Cdc25A physically interacts with ASK1 protein. When Cdc25A is expressed at elevated levels, it suppresses stress-induced activation of ASK1 and downstream kinases [24]. ASK1 mediates the activation of the p38 mitogen-activated protein kinase (MAPK) and the c-Jun NH2-terminal protein kinase-stress-activated protein kinase (JNK/SAPK) following various cellular stresses (Figure 6A) leading to cell death by apoptosis. Since Cdc25A S88F induces cell death of very early embryos and of HT1080 cells after transfection, we tested its ability to suppress ASK1 activity following treatment with a DNA-damaging agent. Human HEK293 cells were transfected with a FLAG vector control plasmid, or plasmids encoding FLAG-Cdc25A-wt or FLAG-Cdc25A S88F, followed by treatment with etoposide (40 μM) for 12 hours. Cell lysates were then probed for immediate targets of ASK-1 mainly SEK and MKK3 as well as their downstream targets, including JNK and caspase 3. Transfected Cdc25A-wt suppressed ASK1 activity as indicated by reduced levels of SEK, MKK3, JNK and caspase 3 (Figure 6B). Cells transfected with Cdc25A S88F cDNA, however, did not suppress ASK1 activity and consequently expression of downstream targets was unaffected. Failure to suppress ASK1 activity translates into activation of apoptosis-mediated cell death. Further analysis showed that Cdc25A S88F interacts poorly or not at all with ASK1 as indicated by its inability to co-immunoprecipitate with the mutant form of Cdc25A (Figure 6C). While FLAG-Cdc25A-wt co-immunoprecipitated with ASK1 from lysates of cells treated with etoposide, FLAG-Cdc25A-S88F failed to do so. These data may explain in part the increased cell death observed in cells that overexpress Cdc25A S88F and in mouse embryos homozygous for the mutant allele.

## Discussion

Cdc25A protein levels are tightly regulated by multiple, apparently redundant, mechanisms. The importance of its tight regulation is underscored by the observation that defects in maintaining steady state levels of Cdc25A translate into increased cell proliferation that can lead to cancer. Indeed, Cdc25A is frequently overexpressed in multiple types of cancer [16]. Ubiquitin-dependent degradation of Cdc25A is a major effector of the DNA-damage-induced S-phase checkpoint. Ubiquitinylation is mediated by the SCF^β-TrCP ^complex which binds to a DSG consensus sequence in Cdc25A that is dependent on phosphorylation of serines 82 and 88 within the DSG motif [10,12].

To begin to dissect the functions of Cdc25A and to better understand the role of ubiquitinylation and degradation in Cdc25A function, we have generated a knock-in mouse (S88F) in which serine 88, one of two key serine residues in the DSG domain, is substituted with a phenylalanine. This serine residue is important for β-TrCP binding and for ubiquitin-dependent degradation of Cdc25A protein. The expectation was that mice expressing Cdc25A S88F would have increased amounts of the protein due to a reduction in its degradation and that cells would undergo unscheduled replication, display genomic instability and early tumor onset in the progeny. No mice that were homozygous for Cdc25A S88F, however, were born. The *Cdc25A*^*-/- *^mice exhibit very early embryonic lethality, which is consistent with a critical Cdc25A function in cell cycle regulation [25]. Analysis of Cdc25A S88F mutant embryos showed that the latest embryonic stage at which homozygous embryos were found was the 3.5 day blastocyst. By this stage, the very few homozygous *Cdc25A *S88F embryos that were present, contained cells that had lost their spherical shape and looked shrunken with blebbed membranes consistent with an apoptotic phenotype. The effect of the *Cdc25A *S88F mutation was apparent at the morula stage and even at the oocyte stage. The rapid cell death that ensued raised a concern that the S88F mutation in Cdc25A produces major conformational changes in the protein or eliminates its phosphatase activity. Analysis of Cdc25A protein structure by CD and assessment of its phosphatase activity *in vivo *and *in vitro *showed that the mutation has only a very minor effect on Cdc25A structure and that its activity is not significantly different than that of wildtype, indicating that neither a change in protein conformation nor a change in enzymatic activity accounts for its lethal phenotype.

Since mutant Cdc25A displays no significant change in protein conformation or phosphatase activity, it is most likely that the lethal phenotype is due to an alternative Cdc25A function. It is known that *Cdc25A *is a transcriptional target of c-Myc and that overabundance of Cdc25A suppresses apoptotic cell death [26,27]. One pathway to apoptosis in which Cdc25A participates involves its interaction with the apoptotic protein ASK1 [24]. When Cdc25A is elevated, it suppresses stress-induced activation of ASK1 and downstream kinases [24]. We therefore tested the possibility that the mutant Cdc25A S88F does not interact with ASK1 and thereby fails to suppress the pro-apoptotic activity of ASK1, promoting apoptotic cell death in early embryos and transfected cells. This scenario is supported by our finding that ectopic expression of wildtype Cdc25A effectively suppresses the expression of targets downstream of ASK1 in response to genotoxic stress while the expression of the mutant protein fails to efficiently do so. In the uterus, embryos are subjected to high levels of reactive oxygen species as a result of the hypoxic environment [28]. Failure of the Cdc25A S88F mutant to suppress ASK1-mediated apoptosis will activate the downstream effectors leading to embryonic lethality.

## Materials and methods

### Generation of Cdc25A S88F mice

Two fragments of Cdc25A genomic DNA were PCR amplified and cloned in a pBSK-TK vector. The first fragment (5030 bp) contained a point mutation GATTC to GATTT (on exon 3) at position 4780. A neomycin resistance marker flanked by loxP sites (LoxP-PGK-Neo-LoxP) was included in the intron 3 immediately after the mutation. A second homologous arm (6715 bp) was cloned after the Neo cassette using conventional molecular biology techniques. The sequence of the targeting construct was validated by direct sequencing. The vector was linearized at a unique Not I site and electroporated into 129/Sv ES cells and neomycin-resistant colonies were screened for correct targeting by Southern blots. Two faithfully targeted ES cell lines were identified, and both were injected into the blastocoel of 3.5-day-old blastocysts which were implanted into the uterus of recipient mice. Chimeras carrying the targeted allele were identified, mated with Black Swiss mice, and agouti progeny were assayed for transmission of the knock-in allele. Mice that were homozygous for the S88F variant were crossed with TgN(EIIa-Cre)C79Lmgd mice (Jackson Laboratories, Bar Harbor, ME) in an FVB/N strain background. Cre recombinase is selectively expressed in the germline of the progeny mice so that their offspring will retain the targeted allele without the *neo *marker but with a single remaining intronic loxP site. All animal experiments were carried out under protocols approved by the Institutional Animal Care and Use Committee of the University of Cincinnati.

### Protein purification and circular dichroism

GST-tagged Cdc25A-wt and Cdc25A S88F proteins were purified using a combination of ammonium sulfate precipitation (65% cut-off), a size exclusion column fractionation, a sulphopropyl (SP) column to separate the GST tag from Cdc25A protein following GST tag removal from the fusion protein using PreScission™ Protease (Sigma), and a glutathione affinity column run according to the manufacturer's protocol (Sigma). Purified wildtype and mutant Cdc25A far-UV CD spectra were measured using an Aviv 215 circular dichroism spectrometer. Proteins were dialyzed into 10 mM Tris pH 8.0, 150 mM NaCl, 1% ethylene glycol, and 2 mM β-mercaptoethanol, and spectra were measured from 260 to 190 nm. Protein concentrations were determined using the absorbance at 280 nm and a molar extinction coefficient of 31,860 M^-1^cm^-1^, as determined by Sednterp [29]. Data were converted to mean residue ellipticity [θ] using the equation $[\theta ]=\frac{\theta }{10*C*l*n_{r}}$ where θ is the measured ellipticity in millidegrees, *C *is the concentration in molar units, *l *is the pathlength in cm, and *n*_*r *_is the number of residues. Data were analyzed using CDSSTR [30] in Dichroweb [31] with reference set 4.

### Cdc25A phosphatase assay

The phosphatase activity of Cdc25A was assayed by hydrolysis of 4-nitrophenol phosphate (pNPP) (Roche Applied Science, Indianapolis, IN) as described previously [32] with modifications. GST-Cdc25A-wt and GST-Cdc25A-S88F mutant were purified from bacteria and then incubated in phosphatase reaction buffer (50 mM Tris pH 8.0, 50 mM NaCl, 1 mM EDTA, 1 mM DTT, and 20 ng pNPP) for 6-10 h at 37°C. The reaction was stopped with 5N NaOH. Cdc25A activity was calculated by measuring the absorbance of *p*-nitrophenolate at 410 nm and subtracting the control background value. Each point is the mean ± SEM of data from two separate experiments.

### Annexin V staining for flow cytometry

Apoptosis was measured using Annexin V-Cy5.5 (Invitrogen, Carlsbad, CA) and propidium iodide (PI). Briefly, harvested cells were washed in phosphate-buffered saline (PBS) and resuspended in Annexin V binding buffer (10 mM Hepes pH 7.4, 140 mM NaCl, 2.5 mM CaCl_2_). Cells (2 × 10^5^) were incubated with 5 μl Annexin V-Cy5.5 and PI (1 mg/ml) for 10 min in the dark at room temperature and analyzed by flow cytometry.

### Immunocytochemistry

NIH3T3 cells were transfected with a GFP-Cdc25A-wt, a GFP-Cdc25A-S88F or a GFP-H2B control plasmid. The cells were washed with PBS 16-18 hours post transfection and fixed with 4% paraformaldehyde. Coverslips were washed with PBS and mounted with Gelmount (Fisher Scientific, Pittsburgh, PA). Fluorescent green cells were then visualized with the use of a LSM510 laser scanning confocal or Orca microscope (Zeiss, Oberkochen, Germany).

## Competing interests

The authors declare that they have no competing interests.

## Authors' contributions

EMB planned and performed the experiments, analyzed the data and wrote the paper. MY, YQL, SBR, DGC and ZY, performed the experiments, RAK, ABH planned and analyzed the data and PJS planned and analyzed the data and wrote the paper. All the authors read and approved the final manuscript.
